# Percutaneous CT-Guided Cryoablation of T1b Renal Cell Carcinoma: A Retrospective Study of Efficacy and Safety

**DOI:** 10.1007/s00270-025-04242-0

**Published:** 2025-11-03

**Authors:** Theresa Junker, Christian Greve Jensen, John Valtersson, Mie Gaedt Thorlund, Tommy Kjærgaard Nielsen, Jens Borgbjerg, Ole Graumann

**Affiliations:** 1 Research and Innovation Unit of Radiology - UNIFY, University of Southern Denmark, Odense, Denmark; 2 Department of Urology, Odense University Hospital, Odense, Denmark; 3 Department of Radiology, Copenhagen University Hospital – Rigshospitalet, Copenhagen, Denmark; 4 Department of Urology, Aalborg University Hospital, Aalborg, Denmark; 5 Department of Clinical Medicine, Aalborg University, Aalborg, Denmark; 6 Clinical Cancer Research Center, Aalborg University Hospital, Aalborg, Denmark; 7 Department of Radiology, Akershus University Hospital, Nordbyhagen, Norway; 8 Department of Radiology, Aarhus University Hospital, Aarhus, Denmark; 9 Department of Clinical Medicine, Aarhus University, Aarhus, Denmark

**Keywords:** Renal cancer, RCC, T1b, Cryoablation, PCA

## Abstract

**Purpose:**

The incidence of renal cell carcinoma (RCC) has risen in recent decades, mainly due to the widespread use of diagnostic imaging. Percutaneous cryoablation (PCA) is minimally invasive, making it favorable if surgery is contraindicated. This study evaluates the oncological efficacy and safety of PCA as a treatment for T1b RCC.

**Material and Methods:**

This retrospective study included 35 patients with T1b RCC treated with PCA. Patients were included if they had at least 3 years of follow-up. Oncological outcomes were analyzed using descriptive statistics and Kaplan–Meier survival curves. Furthermore, estimated glomerular filtration rate (eGFR) changes were described, and postoperative complications were graded according to the Clavien–Dindo classification.

**Results:**

*The primary efficacy* rate was 68.6%, with a median follow-up time of 34.7 months (range: 0–66.5 months), and *the secondary efficacy rate* was 77.1%, with a mean follow-up time of 41.6 months (range: 0–66.5 months). After primary PCA, 17.1% (*n* = 6) were incomplete. Local tumor progression was found in 14.3% (*n* = 5) of patients. The disease-free survival rates at 1, 3, and 5 years were 80.0%, 69.3%, and 60.0%, respectively. Four patients (11.4%) progressed from localized RCC to metastatic disease. The average decline in eGFR was 6.7 mL/min/1.73 m^2^ 1 year after PCA. Complications were observed in 11.4% (*n* = 4) of patients.

**Conclusion:**

This study found that treating T1b RCC with PCA was challenging. Local tumor control rates were low, and the risk for metastatic progression was high. However, PCA demonstrated a commendable safety profile, with few complications and good preservation of kidney function.

**Level of Evidence:**

3, a retrospective cohort study.

**Supplementary Information:**

The online version contains supplementary material available at 10.1007/s00270-025-04242-0.

## Introduction

Renal cell carcinoma (RCC) is a significant clinical challenge, ranking as the 14th most common cancer worldwide in 2020 [1]. Over recent decades, the incidence has increased, mainly due to the widespread use of abdominal imaging, which has led to more frequent detection of incidental and localized RCCs [1]. This shift has intensified the need for effective, minimally invasive treatment options. Percutaneous cryoablation (PCA) has emerged as a well-established alternative to surgery for treating T1a RCC (< 4 cm), particularly in patients considered high risk due to advanced age or comorbidities [2–5]. While radical nephrectomy (RN) or partial nephrectomy (PN) remains the standard of care, PCA offers a nephron-sparing approach with growing technical possibilities, such as the use of multiple cryoprobes and precise tumor targeting [2, 6]. Given these advantages, PCA has potential as a treatment option for larger tumors in the T1b category. However, the evidence supporting the oncological safety and efficacy of PCA in this patient subgroup is still limited; wherefore, current clinical guidelines do not yet recommend PCA for T1b RCC, primarily due to concerns about higher rates of local tumor progression compared to surgical resection [7]. Therefore, further research is warranted to clarify whether PCA should be considered a viable treatment for selected T1b RCC patients. The primary aim of this retrospective study is to contribute by assessing oncological effectiveness, including reporting incomplete ablations, local tumor progression, and advancement to metastatic disease during follow-up after PCA for T1b RCC. Furthermore, secondary outcomes include changes in kidney function, complications, and overall survival. By presenting long-term data, this study can establish the foundation for enhancing treatment strategies and guiding future clinical guidelines.

## Material and Methods

### Study Design and Ethics

The National Data Protection Agency (18/33649) and the National Patient Safety Authority (3–3013-3118/1) approved the study. Due to its retrospective nature, the need for informed consent from the study population was waived. The study was carried out following the *STrengthening the Reporting of OBservational studies in Epidemiology* (STROBE) guideline [8].

### Patient Inclusion and Setting

**Fig. 1:**
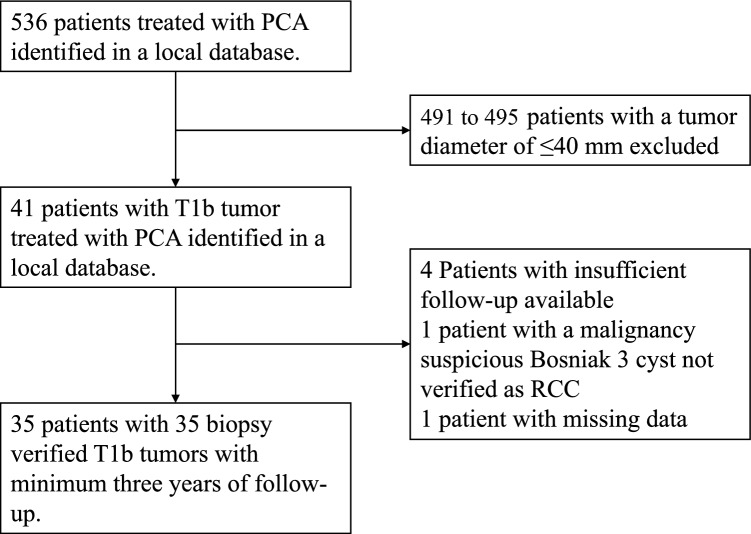
Flowchart of the inclusion process

Patients treated with PCA for RCC with a size of 4.1–7.0 cm (clinical stage T1b) between January 1, 2015, and December 1, 2020, at Odense University Hospital, Denmark, were included. A minimum follow-up of 3 years was required for inclusion in this study. Patient selection was based on a multidisciplinary team conference, which included the participation of urologists, radiologists, oncologists, and pathologists. Recommendations were based on patient comorbidities, including renal function, technical feasibility, and the risk of complications associated with PCA versus PN or RN. Furthermore, patients’ preferences were included based on shared decision-making. All tumors were verified as RCC by renal core biopsy before PCA. Preprocedural imaging of the abdominal and thoracic area was conducted to rule out metastatic disease. A total of 35 patients were included in this study. The process of inclusion is presented in Fig. 1.

Two senior interventional radiologists performed all procedures. A detailed description of the PCA procedure is enclosed as supplementary material (Supplementary material 1).

### Description of Variables

Patient-specific data, including age, sex, weight, height, and comorbidities, were collected from the patient’s electronic medical records. The age-adjusted Charlson Comorbidity Index (CCI) and the American Society of Anesthesiologists (ASA) were applied [9, 10]. Data regarding tumors (size, location, and histopathology) and procedures (probes, hydrodissection, and type of anesthesia) were collected from radiology and histopathology reports. Tumor complexity was determined using the radius-endophytic-nearness-anterior-location (RENAL) nephrometry score and stratified into low (≤ 6), intermediate [7–9], and high [9–11] complexity groups [11]. The hilar suffix was presented separately, not as a subcomponent of the nephrometry scoring system.

*Incomplete ablation* was defined as an enhancement in the ablation cavity detected 3 months after treatment (first follow-up) or if an enhancement was suspected after 3 months and later confirmed. *Local tumor progression* was defined as nodular enhancement in the ablation cavity, as observed in follow-up imaging after the first follow-up scan showed complete ablation. Biopsies were not performed as a standard to confirm local tumor progression or incomplete ablation.

*The primary efficacy rate* was defined as complete ablation of the tumor after the first treatment. *The secondary efficacy rate* was defined as a complete ablation after one or more treatments, per the standardization of terminology and reporting criteria by Ahmed et al. [12]. The follow-up protocol adhered to national guidelines, which involved contrast-enhanced CT (CECT) of the chest, abdomen, and pelvis, or non-contrast chest CT scans, and MRI of the abdomen and pelvis for patients with impaired renal function. These were scheduled at three and 6 months, then annually for 5 years, with repeated CECT scans of the thorax and abdomen, including multiphase contrast imaging of the kidneys. Follow-up data were collected until the December 1, 2023.

Kidney function was evaluated for all patients with available creatinine measurements before PCA, 3 months, and 1 year after PCA. eGFR was calculated using the 2021 CKD-EPI equation [13].

Complications were divided into intraoperative and postoperative complications. Intraoperative complications were defined as complications occurring during the PCA session, and postoperative complications were defined as complications up to 3 months after treatment. Complications were obtained from patient records and imaging reports. Expected symptoms and manifestations after PCA, including pain managed by non-prescription drugs, post-ablation syndrome, and asymptomatic renal hematomas discovered on follow-up scans, were not included as complications as per the standardization of reporting criteria by Ahmed et al. [12]. Postoperative complications were graded using the Clavien–Dindo classification system [14].

### Statistical Analysis

Descriptive statistics were used to summarize patient, tumor, and treatment characteristics. Continuous variables were described as mean with standard deviation (SD) and range or median with interquartile range (IQR) and range, depending on the empirical distributions tested with the Shapiro–Wilk test for normality and by histograms. Categorical variables were presented as frequencies and percentages. Associations between disease progression and tumor characteristics were tested with the Chi^2^ or Mann–Whitney *U* tests based on the variable type.

Kaplan–Meier survival estimate curves were created to depict the time to progression (TTP) and overall survival (OS). Serial times were defined as the time (months) from the primary PCA to the specified event or censoring.

TTP was analyzed as a composite endpoint, including incomplete ablation, local tumor progression, and metastatic disease. Separate Kaplan–Meier analyses were not performed for each event type. Patients were censored at the last available follow-up scan. Consequently, cases of incomplete ablation had a serial time of zero in the Kaplan–Meier estimates, corresponding to a follow-up time of zero.

In OS analyses, death from any cause was considered an event, and patients were censored at the last follow-up date, December 1, 2023.

All analyses were performed using STATA 18 (Stata Corp. 2023, College Station, TX, USA). A *p*-value ≤ 0.05 was considered statistically significant.

## Results

### Patient, Tumor, and Procedural Characteristics

A total of 35 patients were included, with a mean age of 68.6 years (SD 10.71, range 38–86), a median CCI of 4 (IQR 3), and a tumor median of 45 mm (IQR 8, range 41–70). Further details on patient and tumor characteristics are presented in Table 1. A total of 22 patients (63%) were treated using conscious sedation, and hydrodissection was used in 83% (*n* = 29) of procedures. Details on procedural characteristics are presented in Table 2.

**Table 1:** Patient and tumor characteristics of patients (*n* = 35) treated with PCA for RCC T1b

*Patient characteristics*	
Age (years), mean (SD), range	68.6 (10.7), 38–86
Sex *n* (%)	
Male	27 (77)
Female	8 (23)
Body mass index (kg/m^2^), median (IQR), range	27.6 (4.5), 19.1- 45.5
Charlson comorbidity index	
Median (IQR), range	4 (3), 0–10
Low (0–3) *n* (%)	13 (37)
Intermediate (4–6) *n* (%)	15 (43)
High (7–10) *n* (%)	7 (20)
ASA score *n* (%)	
1	3 (9)
2	15 (43)
3	15 (43)
4	2 (6)
Diabetes mellitus *n* (%)	11 (31)
Active smoker *n* (%)	13 (37)
Hypertension *n* (%)	26 (74)
Baseline eGFR^a^ (mL/min/1.73 m^2^), mean (SD), range	76.6 (23.8), 12.7–119.7
*Tumor characteristics*	
Tumor size (mm), median (IQR), range	45 (8), 41–70
RENAL nephrometry score	
Median (IQR), range	9 (2), 5–11
Low (≤ 6) *n* (%)	3 (9)
Intermediate (7–9) *n* (%)	27 (77)
High (10–12) *n* (%)	5 (14)
Histopathology *n* (%)	
Clear cell RCC	27 (77)
Papillary RCC	5 (14)
Chromophobe RCC	2 (6)
Spindle cell RCC	1 (3)

*SD* standard deviation, *IQR* interquartile range, *ASA* American Society of Anesthesiologists, *eGFR* estimated glomerular filtration rate, *RENAL* radius-endophytic-nearness to collection system/sinus-anterior/posterior-locations relative to polar lines

^a^The CKD-epi 2021 formula was used for calculation of eGFR

**Table 2:** Procedural characteristics, based on *n* = 35 primary PCA treatments

Type of anesthesia	
Sedation *n *(%)	22 (63)
General anesthesia *n* (%)	13 (37)
Hydrodissection	
Used in n (%) of treatments	29 (83)
Amount (ml), median (IQR), range	420 (340), 180–1400
Cryoablation probes	
Number mean (SD), range	4.5 (1.1), 2–7
Needle type used in *n* (%) of PCA treatments	
IceForce	26 (74)
IceSphere	8 (23)
IceRod	1 (3)
IcePearl	2 (6)

*IQR* interquartile range, *SD* standard deviation, *PCA* percutaneous cryoablation

### Oncological Outcomes

**Fig. 2:**
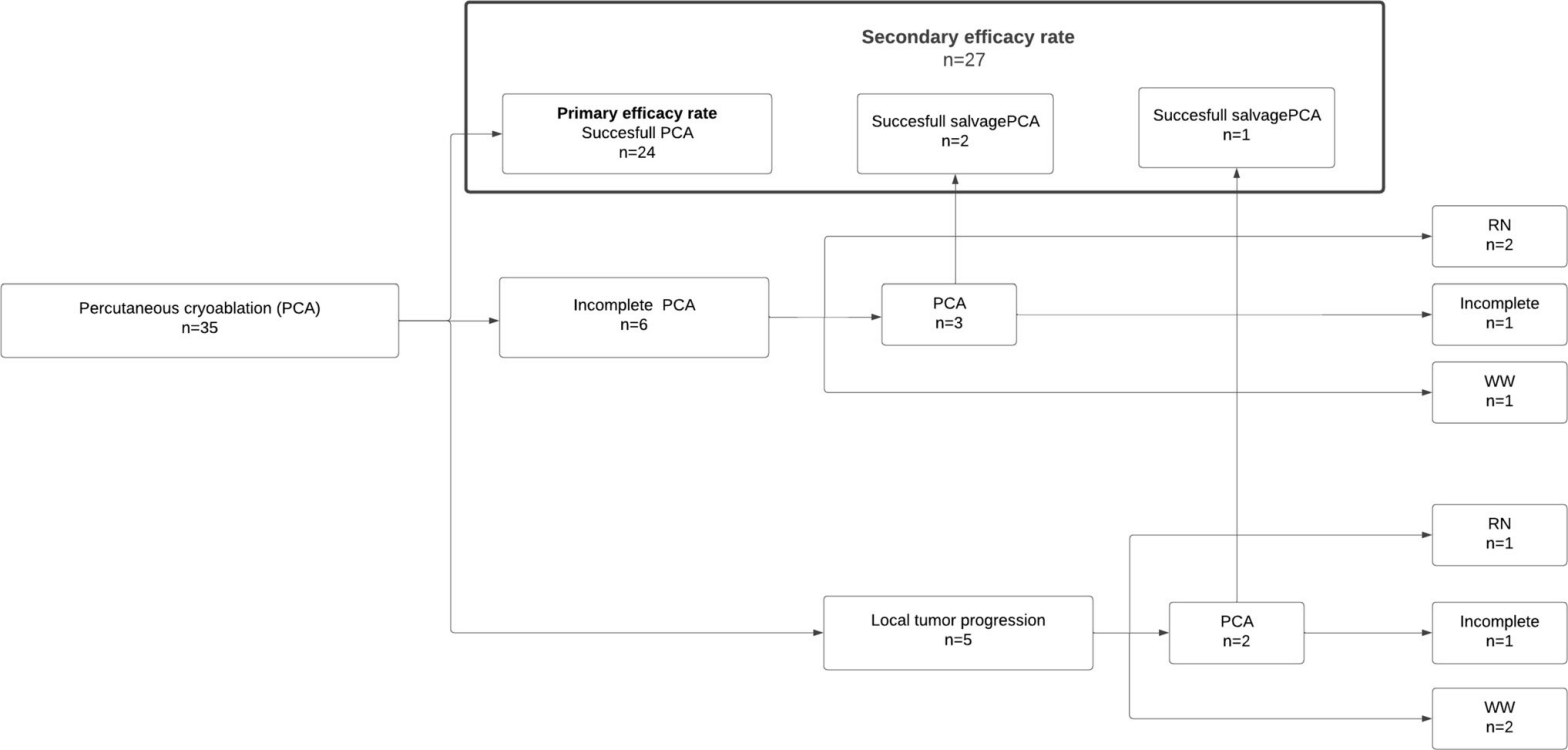
Flowchart of patient treatment

*The primary efficacy* rate was 68.6% (95% CI 0.52–0.82), at a median of 34.7 months (range: 0–66.5). Six procedures were incomplete, of which three received a second PCA; one of these was unsuccessful. Two patients with incomplete ablation received RN, and the last patient continued follow-up with watchful waiting. Furthermore, five patients had local tumor progression during follow-ups at 7, 24, 29, 42, and 47 months, respectively, after primary PCA. Out of these five, two patients had salvage treatment with PCA, one patient had an RN, and two continued follow-ups with watchful waiting. One of the two patients receiving salvage PCA for local tumor progression was unsuccessful and did not receive further therapy except watchful waiting. *The secondary efficacy rate* was 77.1% (95% CI 0.61–0.88), with a median follow-up time of 41.6 months (0–66.5). Figure 2 illustrates the patient treatment flow.

**Fig. 3:**
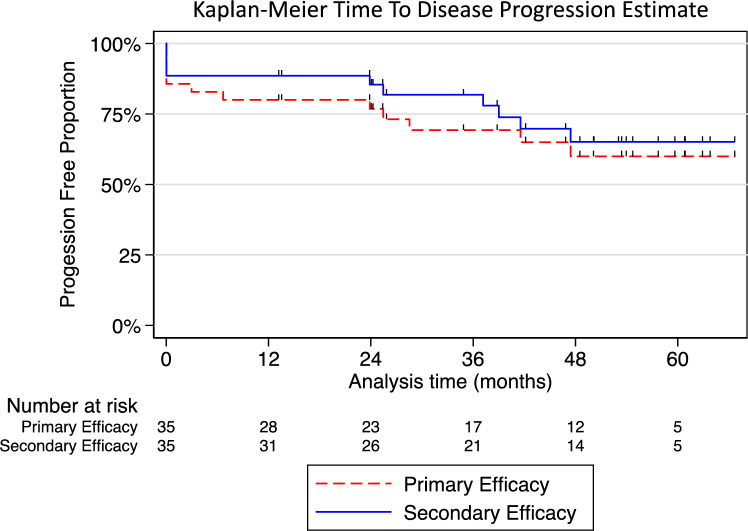
Graphical presentation of the time to disease progression (incomplete ablation, local tumor progression, or progression to metastatic disease) after one PCA treatment

After one PCA procedure, a Kaplan–Meier analysis of TTP showed the proportion of disease-free patients to be 80.0% (95% CI 62.6–89.9), 69.3% (95% CI 50.1–82.3), and 60.0% (95% CI 39.3–75.6) after 1, 3, and 5 years, respectively. When allowing for salvage PCA, the corresponding values were 88.6% (95% CI 72.4–95.6), 81.9% (95% CI 63.8–91.5), and 65.1% (95% CI 44.0–79.9) after 1, 3, and 5 years, respectively (Fig. 3). No tumor characteristics, tumor size (*p* = 0.083), nearness to the collecting system or renal sinus (*p* = 0.784), hilar location (*p* = 0.685), or endophytic tumor growth (*p* = 0.266) were significantly associated with incomplete ablation or local tumor progression. Further details on the oncological outcomes are presented in Table 3.

**Table 3:** Oncological and safety outcomes after PCA of RCC T1b

Treatment status *n* (%)	
Complete	24 (68.6)
Incomplete ablation	6 (17.1)
Local tumor progression	5 (14.3)
Primary efficacy rate	
Proportion local progression free after one treatment n (%, 95%CI)^a^	24 (68.6, 0.52–0.82)
Follow-up time median (IQR), range	34.7 (40.4) 0–66.5
Secondary efficacy rate	
Proportion local progression free after one or more treatments *n* (%, 95% CI)^a^	27 (77.1, 0.61–0.88)
Follow-up time median (IQR), range	41.6 (29.9) 0–66.5
Metastasis *n* (%)	
Metastasis from RCC	4 (11.4)
Treatment of tumor progression* n* (%)	
Salvage cryoablation	5 (14.3)
Watchful waiting/active surveillance	3 (8.6)
Radical nephrectomy	3 (8.6)
eGFR^b^ mL/min/1.73 m^2^	
Three months postoperative^c^ mean (SD), range	65.2 (22.9), 12.7–102.2
Change at three months^c^ median (IQR), range	5.3 (8.9), − 49.0 to 10.8
One year^d^ mean (SD), range	64.9 (24.2), 5.5–116.8
Change at 1 year^d^ median (IQR), range	6.7 (15.1), − 58.5 to 0.6
Complications *n* (%)	
Intraoperative	1 (2.9)
Postoperative	3 (8.6)

*CI* confidence intervals, *IQR* interquartile range, *RCC* renal cell carcinoma, *eGFR* estimated glomerular filtration rate, *SD* standard deviation, *PCA* percutaneous cryoablation

^a^Calculated as Wilson Score Intervals

^b^The CKD-epi 2021 formula was used for calculation of eGFR

^c^Based on 26 measurements

^d^Based on 28 measurements

In total, four patients (11.4%) progressed from localized RCC to metastatic disease. One patient had a metastasis in the colon, which was excised; the patient was alive at the last follow-up. The remaining three patients had metastatic progression to the lungs; of these, one patient received systemic treatment with a complete response.

### Safety

The average decline in eGFR was 6.7 mL/min/1.73 m^2^ 1 year after PCA. Of the nine patients with baseline CKD stage 1 and complete follow-up, five (56%) remained in stage 1, two (22%) progressed to stage 2, and two (22%) progressed to stage 3 (one to stage 3a and one to stage 3b). No patients progressed to stage 4. Additional measures are presented in Table 3.

Complications occurred in 11.4% (*n* = 4) of patients, including one intraoperative complication of an intraprocedural pneumothorax and three postoperative complications: one grade II and two grade IIIb complications, as classified according to the Clavien–Dindo classification. Table 4 presents the characteristics of these complications.

**Table 4:** Short description of intraoperative and postoperative complications, their respective treatment, and Clavien–Dindo classification

Complication	Treatment	Clavien–Dindo
Retroperitoneal hematoma, pain, and macroscopic hematuria	Pain management (opioid and no invasive treatment)	Grade II
Renal hematoma and small abscesses	Antibiotics JJ-catheter	Grade IIIb
Uroplania with connection to hematoma	JJ-catheter	Grade IIIb
Intraprocedural pneumothorax	Inserted chest tube during PCA	N.A

*N.A* not applicable

**Fig. 4:**
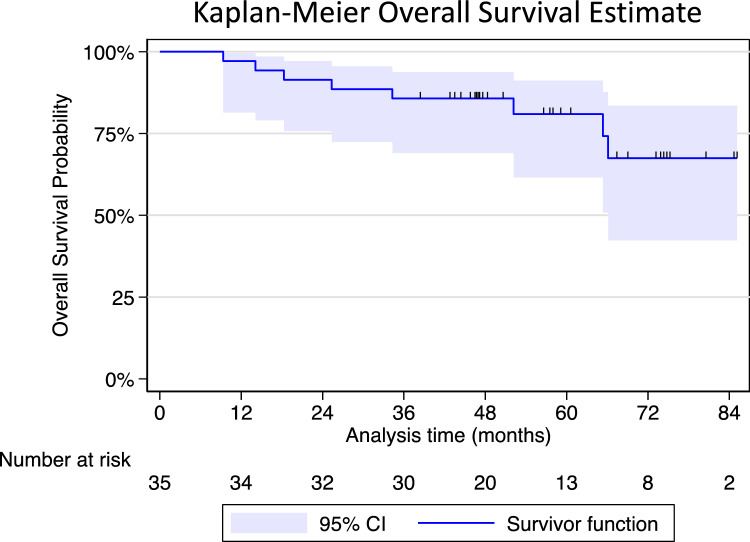
Overall survival from primary PCA until December 1, 2023

Eight patients died during the follow-up period. Two patients had metastatic RCC at the time of death; both patients had substantial comorbidity and died 52 and 66 months after PCA, respectively. The remaining six patients died from other causes not related to RCC or PCA at 9, 14, 18, 25, 34, and 65 months after PCA, respectively, corresponding to a 5-year OS rate of 81% (Fig. 4). Among non-RCC-related deaths, four patients had other malignancies (ages 70–81), one patient had CKD stage 4 and myelofibrosis (age 74), and one patient was in ASA group 3 with complicated diabetes (age 77).

## Discussion

This study found that PCA of biopsy-proven RCC T1b was associated with a secondary efficacy rate of 77.1% and a progression to metastatic disease in 11.4% of cases at a median follow-up time of 41.6 months. The results demonstrate satisfactory measures of local tumor control, consistent with other studies on PCA of T1b tumors [15–18].

### Oncological Efficacy

The present study found that the proportion of disease-free patients after one PCA was 80.0% and 69.3% after 1 and 3 years, respectively. This corresponds with the measurements by Hebbadj et al., which reported 82.6% and 60.5% after 1 and 3 years, respectively [15]. Five patients with local progression were candidates for a salvage PCA, which resulted in corresponding values of 88.6% and 81.9% after 1 and 3 years, respectively. This is essential, given that salvage PCA has been reported to be feasible and safe [19].

Local tumor progression rates vary in PCA studies of T1b tumors and are significantly influenced by follow-up duration, with rates ranging from 2.8% to 23.5% over 13.9 to 26.4 months [15–18]. The present study reported a primary local tumor progression rate of 31.4%. However, if salvage PCA is included, the secondary local tumor progression rate is reduced to 22.9%; both metrics are observed at a longer follow-up time, compared to the studies mentioned above, at a median of 34.7 and 41.6 months, respectively. Salvage treatment with PCA of T1b RCC is supported by Aikawa et al.’s findings of comparable local control rates to PN following salvage PCA for recurrence lesions [20]. Treating T1b RCC with PCA is challenging, as this study shows that 45.5% (5/11) of patients with local tumor progression had it occur more than 3 years after primary ablation. Of these five patients, three underwent salvage PCA treatments but still developed late local tumor progression. Due to the nature of PCA, secure ablative margins cannot be guaranteed, as in the case of extirpative surgery, where the surgeon can be histopathologically informed regarding the resection bed. A small amount of untreated RCC tissue is likely to expand and may only be detected during a late follow-up [21]. This underlines the importance of long-term follow-up, as short follow-up times could overestimate the oncological efficacy of PCA.

The present study found that four patients (11.4%) progressed to metastatic disease after PCA. Generally, metastatic progression rates are low in studies investigating PCA of T1b, ranging from 0 to 9.5% [22]. The extended follow-up time and the small patient population could explain the slightly higher rate of metastatic progression reported in the present study.

Suspected risk factors for disease progression, including tumor size, endophytic properties, and proximity to the collecting system, were tested, but no statistically significant differences were found. However, this lack of significance is likely due to a lack of statistical power. Maxwell et al. examined tumor characteristics and found that a 1-cm increase in size increased the estimated risk of local tumor progression by 198%. Additionally, central placement and proximity to the collecting system significantly increased the risk of tumor progression [23]. Larger tumors are often located near the collecting system, further increasing the risk of local tumor progression. Therefore, tumor size and proximity to the collecting system remain critical considerations when evaluating T1b PCA candidates.

Based on our findings, we therefore recommend extending the surveillance period for T1b RCC patients treated with PCA beyond the standard 5 years. Specifically, annual contrast-enhanced imaging should be continued for at least 7 years post-procedure. This recommendation is based on the fact that nearly half of the local tumor progressions in our study occurred more than 3 years after the initial treatment. Delayed progression may reflect the technical limitations of ablation in achieving reliable oncologic margins in larger or more complex tumors. Therefore, prolonged and vigilant follow-up is essential for the timely detection and management of recurrence in this patient group with T1b RCC.

### Safety

PCA is a nephron-sparing procedure, similar to PN, that offers the preservation of renal tissue, which may reduce the risk of long-term complications, such as chronic kidney disease (CKD). CKD increases the risk of renal failure and cardiovascular disease, resulting in increased overall mortality [2]. Furthermore, CKD has been associated with decreased patient quality of life [24, 25]. These are factors worth considering, given that PCA is often offered to elderly patients with comorbidities or frailty, excluding them from surgery. In this study, eGFR declined by approximately 6.7 mL/min/1.73 m^2^ approximately 1 year after the primary PCA; this is higher but in line with the results found by Grange et al., who reported a decline in eGFR of 4.4 mL/min/1.73 m^2^ [17]. Looking into CKD groups, six patients (17.1%) progressed to a higher CKD group, and two patients progressed more than one group. However, none of the patients with decreased kidney function (CKD 3a or higher) progressed to a higher stage of CKD.

Finally, this study found a complication rate of 11.4% (*n* = 4), with 8.6% (*n* = 3) postoperative Clavien–Dindo grade ≥ II complications. These rates are lower than those reported by Hebbadj et al., who observed a complication rate of 33%, with 11.1% Clavien–Dindo grade ≥ II [15]. Grange et al. reported that 17.4% (*n* = 4) of patients experienced Clavien–Dindo I complications and 4.3% (*n* = 1) experienced Clavien–Dindo II complications [15, 17].

Adjuvant procedures, such as hydrodissection, are widely used to protect adjacent structures during PCA [21]. In this study, 83% (*n* = 29) of primary PCA procedures were performed with hydrodissection, which may contribute to the low complication rate. Furthermore, pre-procedural embolization has been suggested as a supplement to reduce post-cryoablation hemorrhage [26]. However, embolization is not part of the treatment at the institution where this study was conducted. Nevertheless, the complications were comparable to those reported by Atwell et al., who described the systematic use of embolization before PCA in tumors larger than 5 cm [16].

Eight deaths were observed during follow-up in this study, of which two were related to RCC. Both fatalities resulted from metastatic RCC after primary PCA. The 5-year OS rate was 81%, comparable to the 5-year OS rate of 77% reported by Andrews et al. in a sub-cohort of T1b tumors treated with PCA [27]. The OS following surgical treatment, RN versus PN, in RCC T1b is often reported to be higher than the OS reported in the present study [28, 29]. However, a comparison of the populations receiving PN, RN, and PCA introduces the risk of selection bias due to unmeasured confounders.

The retrospective design of the present study introduces a risk of information bias that may have resulted in underreporting of progression or complications, potentially underestimating the actual progression rate. Additionally, incomplete follow-up could lead to overestimation of survival outcomes. However, a rigorous follow-up scheme at the institution where the present study was conducted reduces that risk. Nevertheless, this study contributes to the current literature on PCA treatment of T1b tumors, notably with a relatively long follow-up compared to similar studies. A small sample size limited this study; however, the results gained statistical strength by utilizing a compound endpoint in the TTP analysis. Furthermore, the need for more standardized reporting of outcome variables is evident, making comparisons to existing literature challenging.

In conclusion, this study on PCA for RCC T1b reveals that oncological control is low and the risk for metastatic progression is high. This highlights the inherent challenge in treating this subset of more extensive tumors. Despite this, PCA demonstrated a commendable safety profile, with few complications and good preservation of kidney function. Further research, preferably prospective studies, is needed to confirm the results.

## Supplementary Information

Below is the link to the electronic supplementary material.

Supplementary file1 (DOCX 15 KB)
