# Supplementary material for: Percutaneous CT-Guided Cryoablation of T1b Renal Cell Carcinoma: A Retrospective Study of Efficacy and Safety
Source: Cardiovasc Intervent Radiol. 2025 Nov 3;49(2):301–10. doi: 10.1007/s00270-025-04242-0 (PMC12868034; doi:10.1007/s00270-025-04242-0)
Supplement: Supplementary file 1 — Supplementary file1 (DOCX 15 KB) [file 270_2025_4242_MOESM1_ESM.docx]

**Supplementary Material 1** Description of the cryoablation procedure

In the present institution, experienced interventional radiologists perform percutaneous cryoablation (PCA) in an outpatient setting. Up until September 2017, the majority of PCA seances were performed with the patient under general anesthesia. Hereafter, sedation using Dexmedetomidine, Remifentanil, and an injection of local anesthesia (Lidocaine + Bupivacaine) was more prominently used. All PCA procedures were performed in a Siemens Somatom Flash computer tomography (CT) scanner (Siemens Healthcare, Erlangen, Germany). The type and number of cryoprobes were determined before the procedure to ensure that the size of the ice ball would exceed the tumor tissue sufficiently. The chosen cryoprobes were inserted into the tumor under CT guidance, and an argon-based cryoablation system (IceFx, Boston Scientific, MN, USA) was used. During the procedure, sequential CT scans were performed after four and eight minutes in each freeze cycle to evaluate ice ball formation and needle position and to monitor possible intra-procedural complications. Every cryoablation procedure was performed with a biphasic freeze-thaw cycle, where a standard cycle consisted of 10 minutes of freezing followed by 6.5 minutes of passive and 1.5 minutes of active thawing, ending with 10 minutes of freeze. If the sequential scans showed signs of the tumor not fully encompassed by the ice ball or the ice ball approximating critical tissue, freeze and thaw cycle times were altered accordingly. Anatomical structures adjacent to the treated area were protected by displacement through hydrodissection performed with an 18 cm 18 G percutaneous entry thin wall needle (Cook, Bloomington, IN, USA) with 2% iodine-based saline solution. Following PCA, the patient was observed for up to four hours before discharge.
